# Pauses enhance chunk recognition in song element strings by zebra finches

**DOI:** 10.1007/s10071-015-0855-3

**Published:** 2015-03-14

**Authors:** Michelle Spierings, Anouk de Weger, Carel ten Cate

**Affiliations:** 1 Behavioural Biology, Institute of Biology Leiden (IBL), Leiden University, P.O. Box 9505, 2300 RA Leiden, The Netherlands; 2 Leiden Institute for Brain and Cognition (LIBC), Leiden University, P.O. Box 9600, 2300 RC Leiden, The Netherlands

**Keywords:** Songbirds, Zebra finch, String learning, Chunking, Vocal perception, Song learning

## Abstract

When learning a language, it is crucial to know which syllables of a continuous sound string belong together as words. Human infants achieve this by attending to pauses between words or to the co-occurrence of syllables. It is not only humans that can segment a continuous string. Songbirds learning their song tend to copy ‘chunks’ from one or more tutors’ songs and combine these into their own song. In the tutor songs, these chunks are often separated by pauses and a high co-occurrence of elements, suggesting that these features affect chunking and song learning. We examined experimentally whether the presence of pauses and element co-occurrence affect the ability of adult zebra finches to discriminate strings of song elements. Using a go/no-go design, two groups of birds were trained to discriminate between two strings. In one group (Pause-group), pauses were inserted between co-occurring element triplets in the strings, and in the other group (No-pause group), both strings were continuous. After making a correct discrimination, an individual proceeded to a reversal training using string segments. Segments were element triplets consistent in co-occurrence, triplets that were partly consistent in composition and triplets consisting of elements that did not co-occur in the strings. The Pause-group was faster in discriminating between the two strings. This group also responded differently to consistent triplets in the reversal training, compared to inconsistent triplets. The No-pause group did not differentiate among the triplet types. These results indicate that pauses in strings of song elements aid song discrimination and memorization of co-occurring element groups.

## Introduction

Learning which syllables of a continuous speech stream belong together as words is one of the first challenges that human infants face when they are acquiring a language. In order to do so, infants attend to the pauses between words (Nazzi et al. 2000; Johnson and Jusczyk 2001; Thiessen and Saffran 2003; Lew-Williams et al. 2011). However, pauses are not always reliable and can occur both between and within words. Another way of detecting regularities is by paying attention to the transitions between syllables (Saffran et al. 1996a, b; Aslin et al. 1998). Syllables that occur together more often and have a higher transitional probability are more likely to form a word. Infants also use this feature to correctly segment speech streams. Computational models support the hypothesis that transitional information can be sufficient for correct word segmentation (Brent and Cartwright 1996; Swingley 2005).

Humans are not the only animals that segment longer acoustic sequences into smaller units. Other vocal learners, like songbirds, copy groups of song elements or song types from tutor songs and combine these in their own song. Nightingales, for instance, learn ‘packages’ of a few song types and combine these as units in their own song sequences (Hultsch and Todt 1989a). When exposed to many song types, young nightingales tend to copy groups of song types that often occur together or that are surrounded by longer pauses (Hultsch and Todt 1989b, 2004). This shows that the packages they learn are based on both proximity of song types in the tutors’ song, as well as on pauses between song types.

Songbirds with less vocal variation also show a tendency to copy chunks from their tutors’ song. Zebra finches often copy groups of elements instead of single elements and can combine chunks from different tutors into their own song (ten Cate and Slater 1991; Williams and Staples 1992). In the tutor songs, these chunks are separated by relatively long silent intervals (Williams and Staples 1992). Interrupted songs are terminated most often at the end of chunks and respiratory patterns show inhales and exhales at chunk edges (Cynx 1990; Franz and Goller 2002). Bengalese finches also seem to perceive songs as a composition of chunks (Suge and Okanoya 2010) and combine chunks in their own song (Takahasi et al. 2010). The elements within these chunks co-occur more often and have shorter pauses between them compared to elements of adjacent chunks (Okanoya 2004; Seki et al. 2008; Takahasi et al. 2010). These studies imply that pauses between groups of elements and co-occurrence of elements within a group affect the memorization of songs and song segments in young birds. However, in natural songs, pauses and element co-occurrence often coincide, making it hard to establish the importance of each factor. Also, it is unknown whether pauses and element co-occurrence affect song memorization in adult birds.

In the current study, we examine the role of both pauses and co-occurrence on song memorization in adult zebra finches. To this end, we trained the birds to discriminate artificially edited strings of song elements. Zebra finches are able to pay attention to both position and co-occurrence when learning sequences of elements (Chen and ten Cate 2015) and can identify short strings of identical song elements based on differences in element sequence (van Heijningen et al. 2009, 2013; Chen et al. 2014; ten Cate 2014). In the current experiment, elements in the training strings are arranged in triplets based on co-occurrence, or—in the second experimental group—based on pauses between the triplets as well as element co-occurrence.

## Methods

### Subjects

Twenty-eight Zebra finches (14 males, 14 females; ages 175–280 days post hatching) were used for this study. All birds were bred and reared at Leiden University and had not been used in experiments before. Half of the birds were assigned to the Pause-group, the other half to the No-pause group (seven males and seven females in both groups; age Pause-group: *M* = 217, SD = 30, age No-pause group: *M* = 215, SD = 34). Before the experiment, the zebra finches were housed in single sex groups on a 13.5 L:10.5 D schedule at 20–22 °C. During the experiment, water, grit and cuttlebone were available ad libitum. Food was used as reinforcement and only available after a correct trial. The birds’ food intake was monitored daily and additional food was given when necessary.

### Operant cages

The experimental setup was identical to that used by Spierings and ten Cate (2014). All experiments were conducted in an operant conditioning cage [70 (l) × 30 (d) × 45 (h) cm]. Each cage was in a separate sound-attenuated chamber and illuminated by a fluorescent tube that emitted a daylight spectrum on a 13.5 L:10.5 D schedule. A speaker (Vifa 10BGS119/8) was located 1 m above the cage. The cage was made from wire mesh except for the floor and a plywood back wall which supported two pecking keys with LED lights. A food hatch was located in between these two keys, easily accessible to the birds. Pecking the left key (sensor 1) elicited a stimulus and illuminated the LED light of the key on the right (sensor 2). Depending on the sound, the bird had to peck sensor 2 or had to withhold its response. A correct response resulted in access to food for 10 s and an incorrect response led to 15 s of darkness.

### Training

The experiment consisted of one shaping phase and two training phases. Shaping was required to familiarize the subject with the setup. During the first training phase (string discrimination training), the birds had to discriminate between two strings. The second training phase (segments reversal training) was a reversal training in which the birds were trained on several specific combinations of elements from the initial strings. During this reversal training, the corresponding feedback was reversed; combinations of elements that originated from the go-string were now no-go items and vice versa.

### Shaping

Zebra finches were first trained in the go/no-go task without exposure to the experimental stimuli. The birds received a conspecific song as the go-stimulus and a pure tone as the no-go stimulus. Each day, the discrimination between the stimuli by each individual was calculated as a percentage correct score (%C) as follows: (correct go responses + correct no-go rejections)/total number of trials. If a bird made no mistakes in the discrimination by always responding to a go-stimulus and never to a no-go stimulus, their %C would be 1. For example, in 20 trials, this would be (10 go responses + 10 no-go rejections)/20 trials. Performance *at random* results in an %C of 0.5, for example, when a bird pecked to a go-stimulus in only 50 % of the cases and also to a no-go stimulus in 50 % of the cases over 20 trials: (5 go responses + 5 no-go rejections)/20 trials = 0.5. A fully incorrect discrimination (only responding to no-go stimuli) would lead to a %C of 0. This shaping phase lasted until the zebra finch reached the shaping criterion of %C >0.8 for three consecutive days, after which the training switched to the string discrimination training.

### String discrimination training

All individuals were trained with one go and one no-go string of zebra finch song elements. For every block of 100 trials, the %C was calculated. A bird progressed to the segments reversal training after reaching the learning criterion of eight consecutive blocks of 100 trials with %C >0.8.

### Segments reversal training

The segments reversal training consisted of triplets of elements which were reinforced with reversed contingencies compared to the string training. Segments from the go-string were now reinforced as being no-go stimuli and segments from the no-go string as go-stimuli. For example, a go-response to a triplet from the previous go-string would now result in 15 s of darkness, and a go-response to a triplet from the previous no-go string would result in 10 s of food access. This phase lasted for 3000 trials, independent of the birds’ performance. The hypothesis underlying this reversal training was that the birds would have most difficulties with reversing their response to triplets that had become associated strongly with the feedback during the string training. Overall, animals show an increase in incorrect responses after the contingencies of stimuli are reversed. However, the speed by which these new contingencies are learned might be influenced by several factors (for an overview, see Mackintosh 1969). Therefore, we focus on the first 20 trials after the reversal only.

### Stimuli

#### String stimuli

**Fig. 1:**
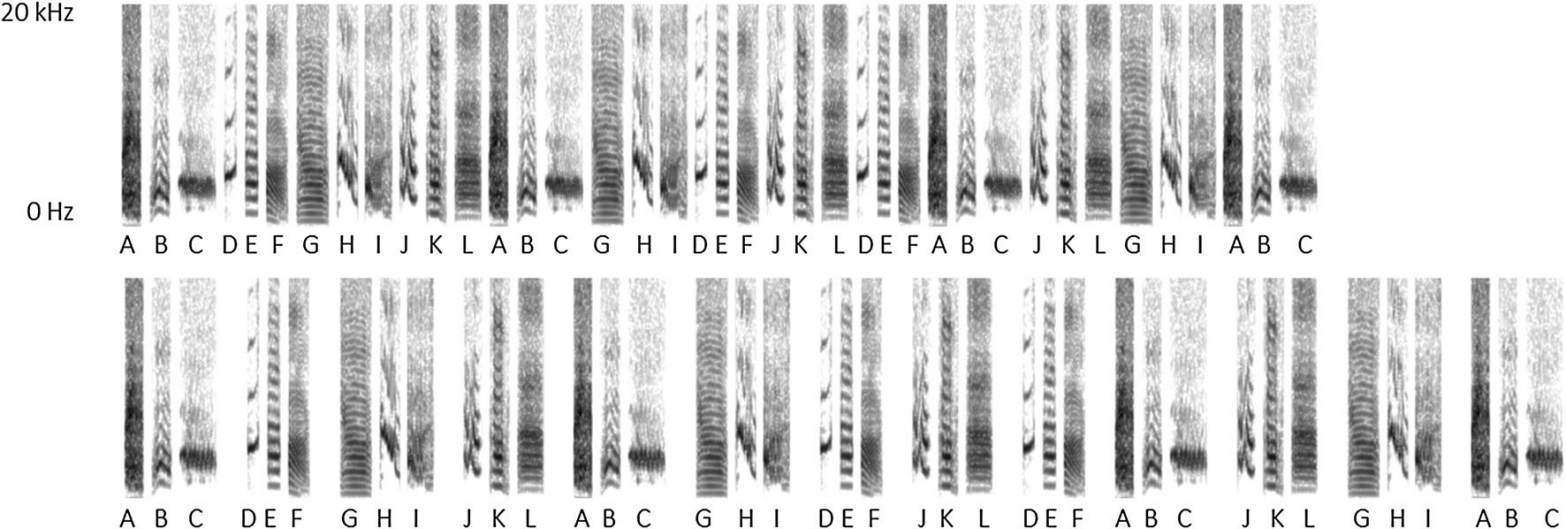
Example of a string with and a string without pauses. Adjacent elements were separated by a 20 ms pause. In the Pause-condition a 80 ms pause separated the triplets

Two strings were constructed from 12 zebra finch song elements, originating from normal songs. All elements were chosen to be from different element categories and were equalized in amplitude, and the beginning and end of the elements were ramped (5 ms on each side) with Praat (Boersma and Weenink 2014). Both strings consisted of four unique triplets, which were a fixed concatenation of three different song elements (for examples of a go and no-go string, see Fig. 1). The triplets of the go and no-go string shared the starting element, but the second and third element of the triplets were different in the two strings (Table 1). The strings were arranged with 20 ms pauses between adjacent elements. The No-pause experimental group was trained to discriminate between two of such strings. The Pause-group was trained using the same strings, but with prolonged pauses (80 ms) between the triplets (Fig. 1). The letters in Table 1 depict the 12 song elements that were used and their order in the string. To avoid pseudo replication, the element represented by each letter was different for each bird within a group. For instance, element ‘A’ could be a ‘high trill’ element for one bird, but would be a ‘flat’ element for another bird. Each string combination occurred both in the Pause-group and the No-pause group, resulting in fourteen different go and fourteen different no-go strings.

**Table 1:** The go and no-go strings as presented to the birds

Go string												
ABC	DEF	GHI	JKL	ABC	GHI	DEF	JKL	DEF	ABC	JKL	GHI	ABC
No-go string												
AFK	DLB	GIE	JCH	AFK	GIE	DLB	JCH	DLB	AFK	JCH	GIE	AFK

Each string consisted of four different triplets, here shown with pauses between the triplets. The No-pause group received the elements in a continuous string. By organizing the triplets in this way, we created strong co-occurrence of song elements within a triplet and lower co-occurrence of elements of different triplets. The letters represent zebra finch song elements which were different for each individual in a group

#### Segment stimuli

Three different types of triplets were created for the reversal training, which we refer to as consistent triplets, partly consistent triplets and inconsistent triplets (Table 2). 1) Consistent triplets were a combination of three elements that had always occurred as a concatenated triplet in the training string. This means that these three elements had high co-occurrence and occurred in both experimental conditions without long pauses between them. 2) Partly consistent triplets were a combination of the last element of one triplet and the first two elements of the following triplet. This means that the co-occurrence of the first and the second element of a partly consistent triplet is lower than the co-occurrence of the second and third element. In the Pause-condition, there had also been a pause between the first and second element of a partly consistent triplet in the initial training strings. 3) Inconsistent triplets consisted of a combination of elements that had never occurred together during the string discrimination training. Therefore, there had not been co-occurrence between these elements in the training strings. For each triplet category (consistent triplets, partly consistent triplets and inconsistent triplets), two triplets were derived from the go-string and two from the no-go string of each bird (see Table 1 for a representation of the string stimuli, and Table 2 for a representation of the consistent triplets, partly consistent triplets and inconsistent triplets).

**Table 2:** The different triplets created for this training

	From go-string		From no-go string	
Consistent triplets	JKL	GHI	JCH	GIE
Partly consistent triplets	KLD	EFG	CHD	LBG
Inconsistent triplets	ELH	KID	LEC	EBJ

These triplets were different combinations of the elements from the string discrimination training

### Statistical analyses

#### String discrimination training

This training phase was completed when an individual reached a %C > 0.8 for eight successive blocks of 100 trials. The number of days and the number or trials needed to reach this criterion were measured for each individual. These measurements followed a normal distribution (number of trials after a log transformation), allowing us to analyze the results of the Pause-group and the No-pause group in a paired Student’s *t* test. The groups were paired based on the similarity of the discrimination training strings. Every element combination was present once with pauses between the triplets and once without pauses.

#### Segments reversal training

For each individual, an average %C was calculated for the first 20 trials of each triplet category (consistent triplets, partly consistent triplets and inconsistent triplets). These scores were used to measure the first responses of the birds to these segments. The data were analyzed in a linear mixed effects model (LMNE) with %C as the dependent variable and condition (pauses or no pauses) and triplet category as independent variables. Individual was inserted as the random variable. Differences between the triplet categories were exposed with a post hoc Tukey test. Furthermore, we analyzed whether the %C of each triplet category per group deviated from random performance (%C = 0.5), with a one sided *t* test. The significance levels were corrected with a Bonferroni correction due to repeated analyses within one experimental group.

#### Correlations

In order to reveal possible correlations between the first and the second training phase, we ran a Pearson correlation test between the birds’ %C of the three different triplet categories and the number of days and trials they needed to complete the first training phase.

## Results

### String discrimination training

**Fig. 2:**
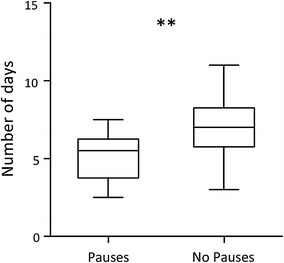
Number of days needed to reach the learning criterion. Discrimination between strings was made more quickly with pauses between the triplets of the strings. The *boxplots* show the median (*horizontal line*) and first and third quartile of the data, with *whiskers* extending to the minimum and maximum values. *Asterisks* indicate a significant difference between the groups

All zebra finches reached the discrimination criterion (3 days with %C >0.8) within 11 days, with an average of 477 trials per day (SD = 109.6). The Pause-group achieved the discrimination earlier than the No-pause group (Pause: *M* = 5.14, SD = 1.61; No-pause: *M* = 7.07, SD = 2.07; *t* = −3.49, *P* = .004; Fig. 2). The Pause-group also needed fewer trials to reach the learning criterion (Pause: *M* = 2485, SD = 718; No-pause: *M* = 3171, SD = 1148; *t* = −2.23, *P* = .03).

### Segments reversal training

**Fig. 3:**
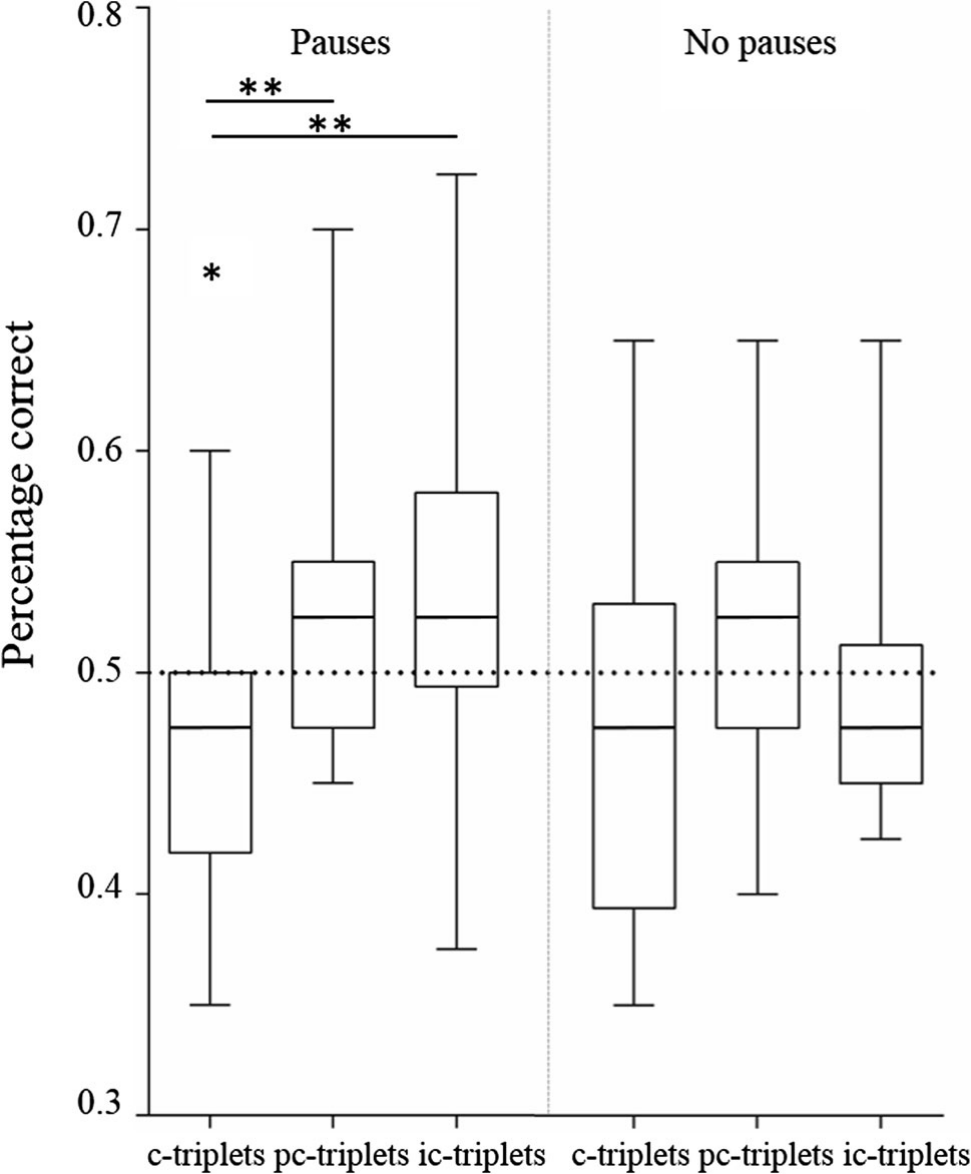
Learning during the first 20 trials per triplet category. Only when the zebra finches that heard pauses in the first training strings responded differently to triplets of co-occurring elements (consistent triplets) compared to triplets of less co-occurring (partly consistent triplets) or not co-occurring (inconsistent triplets) elements. There was no effect of co-occurrence of elements in the condition without pauses. The *boxplots* show the median (*horizontal line*) and first and third quartile of the data, with *whiskers* extending to the minimum and maximum values. *Asterisks* *with a* *line* indicate a significant difference between the groups, the *single asterisk* indicates a significant difference from random (=0.5)

For each triplet category, the %C was calculated over the first 20 trials of the reversal training. The Pause-group responded differently to the consistent triplets (c-triplets) than to the partly consistent triplets (pc-triplets) and inconsistent triplets (ic-triplets) (lmm Tukey c-triplets vs. pc-triplets: *z* = −3.01, *P* = .007; c-triplets vs. ic-triplets: *z* = −3.51, *P* = .001; pc-triplets vs. ic-triplets: *z* = −0.50, *P* = .87; Fig. 3). The response to consistent triplets was significantly lower than random (=0.5), while the responses to both partly consistent triplets and inconsistent triplets did not deviate from random (c-triplets: *M* = 0.46, SD = 0.06, *P* = .04; pc-triplets: *M* = 0.53, SD = 0.06, *P* = .17; ic-triplets: *M* = 0.54, SD = 0.08, *P* = .13). The No-pause group showed no difference in response to consistent triplets, partly consistent triplets or inconsistent triplets (lmm Tukey c-triplets vs. pc-triplets: *z* = −1.48, *P* = .30; c−triplets vs. ic-triplets: *z* = −0.74, *P* = .74; pc-triplets vs. ic-triplets: *z* = 0.74, *P* = .74; Fig. 3). Neither of the groups showed an effect of sex (pauses: *P* = .43; no pauses: *P* = .87).

**Fig. 4:**
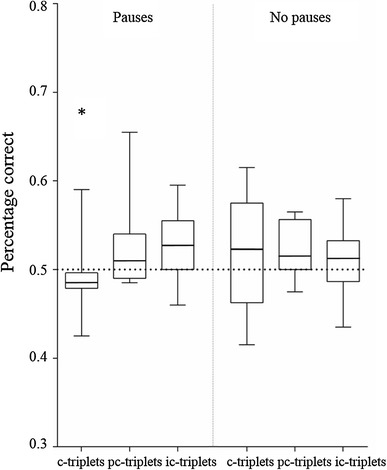
Results of the first 100 trials per triplet category. Zebra finches that heard pauses in the string discrimination training made more incorrect responses to the consistent triplets compared to the partly consistent triplets or inconsistent triplets. There was no difference in response between the triplet categories in the No-pause group. The *boxplots* show the median (*horizontal line*) and first and third quartile of the data, with *whiskers* extending to the minimum and maximum values. *Asterisk* indicates a significant difference from random (=0.5)

These results remained consistent over the first 100 trials of each triplet category. In the Pause-group, the responses to the consistent triplets stayed lower than the responses to the partly consistent and inconsistent triplets (lmm Tukey c-triplets vs. pc-triplets: *z* = −2.70, *P* = .02; c-triplets vs. ic-triplets: *z* = −3.14, *P* = .005; pc-triplets vs. ic-triplets: *z* = −0.44, *P* = .90; Fig. 4). The No-pause group continued to not show a difference in response to any of the triplet categories (lmm Tukey c-triplets vs. pc-triplets: *z* = −1.16, *P* = .478; c-triplets vs. ic-triplets: *z* = −0.33, *P* = .94; pc-triplets vs. ic-triplets: *z* = 0.83, *P* = .68; Fig. 4).

There was no difference in response to one of the two consistent triplets, one of the two partly consistent triplets or one of the two inconsistent triplets, except for the partly consistent triplets in the No-pause group, which responded better to the triplet that had occurred earlier in the string (mean %C 1 = 0.58, mean %C 2 = 0.48, *P* = .01), meaning that in this case the location of the elements in the discrimination string influenced the recognition of this particular segment. No significant correlation was found between the duration of the discrimination training and the results in the segments reversal training (Pearson %C triplets with training days = −0.25).

## Discussion

The results of the string discrimination training indicate that zebra finches discriminated more readily between two strings when there were longer pauses between element triplets. It cannot be excluded that this enhanced discrimination is affected by the total duration of the strings, which increases with increased pause length. This means that the birds are exposed to a lower number of elements per unit of time. However, given that longer inter-element pauses in natural songs are more likely to be perceived as a break in a song string, a slower succession of elements seems unlikely to result in a better memory of which elements are to be followed by which others. Rather, and in-line with the finding of segments reversal training, we suggest that the pauses aid in detecting co-occurring element triplets and that this improves learning of strings consisting of such triplets. This interpretation is in-line with the results of many studies showing that strings organized in chunks, from telephone numbers for humans to sequences of visual tokens to be learned by pigeons or rats, are memorized faster and better than strings with an equal number of items providing no chunking cues (e.g., Terrace 1987; Terrace and Chen 1991; Fountain et al. 2012).

The results of the segments reversal training demonstrate that pauses in strings of song elements elicited an enhanced memorization of co-occurring element triplets that were surrounded by pauses. The mere co-occurrence of song elements in strings without pauses did not evoke a similar response, as demonstrated by the lack of an association between triplet category and %C in the reversal training of the No-pause group. This indicates that pauses between chunks positively affect the memorization of these chunks.

In their natural songs, zebra finches produce longer pauses between and shorter pauses within chunks (Zann 1993). We suggest that the natural longer inter-chunk pauses enhance song memorization in young birds and might therefore play an important role in the song-learning process (Hultsch and Todt 1989a, b; ten Cate and Slater 1991; Williams and Staples 1992; Suge and Okanoya 2010; Takahasi et al. 2010). Moreover, because our experiment was conducted with adult zebra finches and individuals of both sexes, we show that this proposed learning advantage of the presence of longer inter-chunk pauses is not specific for the period of song production learning. The zebra finches also memorized chunks of co-occurring elements better with longer pauses between such chunks, even though their song learning phase had finished. This suggests that under natural conditions, the presence of such pauses may help adult birds to memorize the songs of different individuals with rather similar songs and hence may help to discriminate among individuals. Bengalese finches, a related vocal learning species, use either co-occurrence, longer pauses or both when they are copying parts of songs from their tutor (Takahasi et al. 2010). In natural songs, as used in the aforementioned experiment, these two factors are strongly correlated. The results from our study show that zebra finches are more sensitive to these pauses than to co-occurrence. This could be an indication that when songbirds have two correlating cues that they can use, like co-occurrence and pauses, they might be more prone to pay attention to the pauses.

Although co-occurrence of elements on its own did not create better recognition of element groups by zebra finches in the present experiment, an earlier study (Chen and ten Cate 2015) showed that zebra finches can use co-occurrence for sequence discrimination and also remember co-occurring items when co-occurring elements are re-shuffled in position within sequences. The birds could use similarity in both transitional and positional information of the training strings to discriminate between new strings. Interestingly, the zebra finches responded to the most reliable cue in that particular context, indicating a context-dependent learning strategy. Apart from songbirds, other animals can also respond to element co-occurrence. Tamarins (*Saguinus oedipus*) and rats (*Rattus rattus*), for instance, both respond more strongly to segments of a string with high co-occurrence (Hauser et al. 2001; Toro and Trobalon 2005). Neither species are considered to be vocal learners, demonstrating that a tendency to attend to co-occurrences is not specific to language or song learning.

In the string discrimination training of the present study, zebra finches of the No-pause group could have used both transitional (co-occurrence) and positional information to make the discrimination. Knowing, however, that they do not differentiate among the different types of triplets, it is likely that the zebra finches made the discrimination using the position of the elements (similar to some of the birds in Chen and ten Cate 2015). This indicates a learning strategy comparable to visual sequence learning in other species (Terrace 1987; Brannon and Terrace 1998; Fountain and Benson 2006). In these studies, animals used positional and ordinal information to memorize the sequential organization of items. Moreover, tamarins and rhesus monkeys were able to learn sequences that could not be chunked and responded above chance to all two-item combination from these sequences. This is an indication that, unlike pigeons and rats, monkeys formed an ordinal representation of the sequences (Swartz et al. 1991; Ohshiba 1997). Likewise, chunking of longer sequences might be a useful tool in memorizing conspecific songs in zebra finches.

Human infants also tend to use pauses as a cue to find word boundaries (Nazzi et al. 2000; Johnson and Jusczyk 2001; Thiessen and Saffran 2003; Lew-Williams et al. 2011). When pauses are present in a speech stream, infants tend to treat inter-pause segments as more familiar than segments that span pause boundaries. This is quite similar to the responses of the zebra finches. However, human infants are also able to use co-occurrence or transitional probabilities between elements when pauses are not a reliable cue (Saffran et al. 1996a, b; Aslin et al. 1998). Although zebra finches are able to use transitional probabilities (Chen and ten Cate 2015) and might use them during song learning (Lipkind et al. 2013), the current experiment shows that they do not readily form an association with chunks formed by co-occurrence only.

In conclusion, longer pauses between chunks in strings of song elements aid zebra finches in the song recognition process. These pauses also stimulate memorization of segments of strings that are determined by such pauses. The co-occurrence of song elements on its own does not elicit similar learning advantages. These results indicate that pauses between chunks of song elements might function not only as an aid to song learning in juvenile birds, but also to song discrimination in adult birds of both sexes.
